# Evaluation of the Inter and Intra-Observer Reliability of the AO Classification of Intertrochanteric Fractures and the Device Choice (DHS, PFNA, and DCS) of Fixations

**DOI:** 10.4314/ejhs.v30i5.15

**Published:** 2020-09

**Authors:** Mohamed Zarie, Mohamed Farah Mohamoud, Amir Reza Farhoud, Nima Bagheri, Furqan Mohammed Yaseen Khan, Mahdi Heshmatifar, Hadi Klantar

**Affiliations:** 1 Department of Orthopedics, School of Medicine, Tehran University Medical Sciences, Tehran, Iran; 2 International Campus, Tehran University Medical Sciences, Tehran, Iran

**Keywords:** Osteosynthesefragen classification, Intertrochanteric fracture, Reliability, Orthopedic trauma

## Abstract

**Background:**

ArbeitsgemeinschaftfürOsteosynthesefragen (AO) classification is the most frequently used tool to classify intertrochanteric fractures. However, there is limited evidence regarding its reliability. Therefore, this study was designed to evaluate inter-observer and intra-observer reliability of the AO-2018 intertrochanteric fracture classification.

**Method:**

A retrospective study was conducted in Imam Khomeini Hospital Complex, on radiography of patients who came with intertrochanteric fractures from March 21, 2018, to March 19, 2019. Four orthopedic trauma surgeons assessed 96 anteroposterior pelvic radiographs of intertrochanteric fractures and classified using an AO intertrochanteric fracture classification of 2018. The reading and review of radiography were performed in 2 separate occasions in a 1-month interval. The inter-observer and intra-observer reliability was assessed using kappa statistics.

**Result:**

The level of both mean inter-observer (K =0.322; 95%CI: 0.321–0.323) and intra-observer agreement (K =0.317; 95%CI: 0.314–0.320) in AO intertrochanteric fracture classification subgrouping were not satisfactory. The inter-observer (K =0.61; 95%CI: 0.608–0.611) and intra-observers' (K=0.560; 95%CI: 0.544–0.566) reliability in AO main groupings showed moderate agreement.

**Conclusion:**

The AO classification does not show adequate and acceptable inter-observer and intra-observer reliability and reproducibility. Therefore, it will be hard to base on the AO classification for treatment protocols.

## Introduction

Intertrochanteric fracture is the fracture that occurs in the region between greater and lesser trochanters of the proximal femur. It is extracapsular where the vascularity of the femoral head is rarely affected (1). Intertrochanteric fracture makes about 50% of the hip fractures which is caused by low energy mechanisms such as falls (2). It can occur in both the elderly and the young. However, it is more common in the elderly population with osteoporosis due to low energy mechanisms (3). Generally, 6 million hip fractures are estimated to occur by 2050 (4).

Most of the patients present with the absence of weight-bearing, painful shortened, and externally rotated lower limbs (5). For the evaluation and diagnosis of intertrochanteric fractures, standard X-ray of the pelvis and femur can be used. The radiological finding can also help to measure the width of the medullary cavity and assessment of the diaphyseal morphology. Thus, adequate radiological evaluation is required to understand fracture type, and for preoperative planning (6).

The primary goal of intertrochanteric fracture treatment is the early mobilization and avoidance of secondary complications which can be achieved by appropriate reduction and fixations through different fixation devices (7). There are a number of fixation devices available for the treatments. Each has its indications, advantages, and disadvantages. The selection of the devices depends on the type of fracture. However, no implant fully satisfies all fixation requirements of intertrochanteric fractures (8). Implant selection and placement are important factors that can determine and predict the failure of fracture after fixation (9). Identifying the presence of atypical fractures or unstable fracture patterns is important for fracture management (10). The classification system should be valid and reliable and should have a prognostic value that can assist us to plan treatment protocols (11).

As the AO classification is the most commonly used classification that is utilized to base our protocols of choosing appropriate fixation devices, it is worth much to assess the reliability, to optimize the treatment outcome. Therefore, this study aimed to evaluate the inter-observer and intra-observer reliability of the AO classification system in intertrochanteric fractures.

## Methods

This retrospective study was conducted in Imam Khomeini Hospital Complex, Tehran, Iran. Patients with an intertrochanteric fracture who were admitted to the hospital from March 21, 2018, to March 19, 2019, were included in this study. All adult patients (age ≥ 18 years) with new intertrochanteric fractures were included. However, patients with pathological fractures, periprosthetic fractures, and subtrochanteric and neck fractures were excluded. Initially, the radiographs of 136 patients were identified, but 96 of the 136 radiographs met our inclusion criteria and were enrolled in this study. Four orthopedic trauma surgeons had evaluated and read the radiographs (x-ray) findings and provided their classifications twice in the onemonth interval.

**Data sources and collection**: The health information system (HIS) of Imam Khomeini Hospital Complex was used to identify patients with an intertrochanteric fracture in the data collection period. The demographic characteristics of the patients such as age and sex, and the radiologic image (X-ray) were obtained from the HIS. The X-rays were matched and coded with the structured questionnaires. Four experienced orthopedic trauma surgeons who conduct on an average per month 4–7 intertrochanteric fracture fixations independently and who had 3 to 10 years of experience reviewed the radiographs and suggested fixation devices type. The radiographs were reviewed at 2 different times with a one-month interval between the readings. The reviewers were not told that there would be a second time reading. For the first round, the observers classified the fracture according to the AO intertrochanteric fracture classification-2018 and suggested their choice of treatment. One month later, the observers were provided with the same set of radiographs rearranged in a different order along with the same questionnaires and a chart of AO Intertrochanteric fracture classification -2018 were asked to classify the fracture and select the suitable treatment fixation of choice.

**Data analysis**: Kappa statistics was performed by SPSS version 24 to assess inter-observer and intra-observer reliability. Interobserver agreement was evaluated by comparing the responses of 4 different observers in 2 different readings, while the intra-observer reliability was evaluated by comparing each observer's reading on 2 different occasions. The kappa value indicates −1.0 (complete disagreement), 0 (chance agreement) and 1.0 (complete agreement). Interpretation of the strength of agreement determined with the kappa values was given by adopting the criteria of Landis and Koch (12). Landis et al classify the level of agreements into six groups: perfect agreement (K ≥ 0.80), substantial agreement (K = 0.61–0.80), moderate agreement (K = 0.41–0.61), fair agreement (K = 0.21–0.41), slight agreement (K = 0–0.21) and poor agreement (K < 0). The level of significance was set at P-value < 0.05.

**Ethical consideration**: This study was ethically approved by the research ethics board of Tehran University of Medical Sciences and obtained approval number ID IR.TUMS.IKHC.REC.1397.257.

## Result

**Patients' characteristics**: A total of 96 confirmed radiological cases of intertrochanteric fractures of both sexes were included in this study. Of these, 52 patients were males. The mean (±SD) age of the participants was 70.5 (±17.3) years with the age range between 20–97 years old. Of the total patients, 47 were in the age category of 60 to 79 years (Table 1).

**Table 1:** Sex and age distribution of patients

Demographic variables		Frequency (%)
Sex	Male	52 (54.2)
	Female	44 (45.8)
Age	20–29	11(11.5)
	40–59	5(5.2)
	60–79	47(49.0)
	≥80	33(34.4)

**Interobserver reliability**: In the first evaluation, interobserver agreement across the 4 observers in AO subgrouping classifications was fair agreement [K= 0.352; 95% CI: (0.351 – 0.353)] (Table 2). The reviewers' agreement regarding AO main grouping in the first round was substantial agreement [K=0.625; 95% CI: (0.623–0.626)]. In the second evaluation, the result showed fair and moderate agreement for both AO subgrouping [K= 0.292; 95% CI: (0.291–0.293)] and AO main grouping [K=0.595; 95% CI: (0.593–0.597)]. The level of agreement among the observers based on the fixation device choice for the first evaluation was moderate agreement [K = 0.560; 95% CI: [0.558– 0.563)], and in the second evaluation, it was fair agreement [K =0.490; 95% CI: (0.488–0.493)] (Table 2).

**Table 2:** Interobserver variation among orthopedic trauma surgeons in AO classification and device fixation of intertrochanteric fractures

Inter-observer variation		Kappa (95% CI)	P-value
First round	AO subgrouping	0.352(0.351–0.353)	<0.001
	AO main grouping	0.625 (0.623–0.626)	<0.001
	Device fixation	0.560 (0.558–0.563)	<0.001
Second round	AO subgrouping	0.292 (0.291–0.293)	<0.001
	AO main grouping	0.595 (0.593–0.597)	<0.001
	Device fixation	0.490 (0.488–0.493)	<0.001

**Intra-observer reliability**: The intra-observer agreement and reliability were assessed and analyzed by comparing the data of the first observation with the data of the second observation. The mean intra-observer agreement for AO subgrouping for all observers was fair [K =0.317; 95% CI:(0.314–0.320)], (Table 3). The mean intra-observer agreement of AO main grouping for observers showed moderate agreement [K=0.560; 95% CI:(0.544–0.566)] (Table 3).

**Table 3:** Mean intra-observer variation among orthopedic trauma surgeons in the AO classification of intertrochanteric fractures

Observations	Kappa (95% CI)	P-value
AO subgrouping	0.317 (0.314–0.320)	<0.001
AO main grouping	0.560 (0.544–0.566)	<0.001

**Inter-observer reliability based on the treatment of choices**: The interobserver agreement based on choice of fixation devices showed moderate agreement at first and second observations (first observation, [K= 0.560; 95% CI: (0.558–0.563)] and (second observation [K= 0.490; 95% CI: (0.488–0.493)]. The mean intraobserver agreement based on the choice of fixation devices also showed a moderate level of agreement [K=0.560; 95% CI:(0.544–0.566)].

## Discussion

In this study, several attending orthopedic trauma surgeons who had different levels of experience in terms of intertrochanteric fracture management participated to evaluate the reliability of the AO 2018 intertrochanteric classification. Classification of intertrochanteric fracture serves as a guideline for treatment and helps to predict the result (13) or provides a reasonable estimation of the likely outcome (14). Therefore, the reliability of the fracture classification depends on the inter-observer and intra-observer agreement. A low level of agreement among and between observers can limit the use of classification systems in decision making (15). If the preoperative classification is not correct, the usefulness of the prognosis will also be limited (14). However, there is limited evidence in the reliability of fracture classification using the AO-2018 classification criteria in the study area. Therefore, this study was intended to determine whether the reliability of the fracture classification depends on the inter-observer and intra-observer agreement.

In this study, the inter-observer reliability in AO intertrochanteric fracture classification for the subgroup analyses of the first and second observations was fair. However, the interobserver reliability in AO intertrochanteric fracture classification for the main group at the first and second observations was moderate. The interobserver agreement based on the choice of fixation devices had also shown moderate agreement at the first and second observations. The intra-observer agreements in the sub and main groupings had shown lower agreement compared to interobserver agreements. The agreements were fair for the subgrouping and moderate for the main groupings.

A previous study reported by Schipper et al (16) which used the AO classification system to classify trochanteric fractures of 20 X-rays indicated a mean intra-observer kappa value of 0.48 and interobserver kappa values of 0.33 and 0.34 in sub-grouping. However, for the main grouping classifications, intra-observer kappa value was 0.78, while interobserver kappa values were 0.67 and 0.63. These findings are in agreement with our results. However, the intra-observer agreement of our study was slightly lower than the interobserver agreement in comparison to the above study (15). Besides, our study evaluated the agreement among observers based on device choice of fixations which showed a moderate level of agreement.

A study reported by Pervez et al (13) in which 88 sets of radiographs were observed by using AO classifications and Jensen modification of the Evans indicated that the mean intra-observer agreements were K = 0.42 for sub-grouping and K = 0.72 for main grouping. Similarly, mean interobserver agreements were K = 0.33 for sub-grouping and K = 0.62 for main groupings. Moreover, a study reported by De Boeck (17) was also found the AO classification unreliable. Our results are in agreement with this study as there is no adequate reliability.

The study reported by Newey et al (18) found that the AO intertrochanteric fracture classification system is unnecessarily complicated and falls short of playing a useful role in the management of intertrochanteric fractures. Since the classification system intends to indicate the nature of the injury and provides a rationale for treatment (18) and most of the orthopedic surgeons use this classification for choosing appropriate fixations or devices, there is the need for modified criteria or classification system which can help the surgeons to make appropriate clinical decisions. This study's main limitation was the use of X-rays which were not equally standardized.

In conclusion, this study of AO intertrochanteric classification did not show adequate acceptable interobserver and intraobserver reliability and reproducibility. Therefore, based on the findings of this study and that of other studies, there is a probability that AO intertrochanteric classification cannot help to support the exact treatment selection protocols since the results were not reliably strong. Finally, it is better to have back up of one extra fixation device (DHS+PFNA or DHS+DCS) during the operation because based on the above results during the operation, a fracture may not become the one which was seen in the radiographic X-ray.

## Figures and Tables

**Figure 1:**
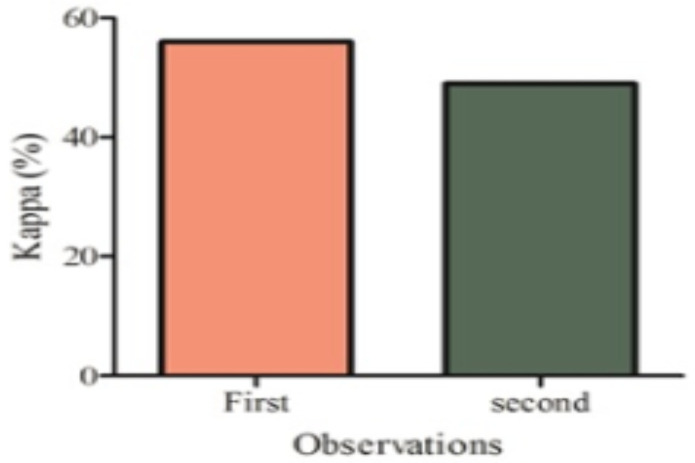
Interobserver agreement based on the treatment of choice at first and second observations.

## References

[1] Egol KA, Koval JK, Zuckerman J (2015). Handbook of Fractures.

[2] Ahn J, Bernstein J (2010). In Brief: Fractures in Brief: Intertrochanteric Hip Fractures. Clinical orthopaedics and related research.

[3] Attum B, Pilson H (2019). Intertrochanteric Femur Fracture.

[4] Kannus P, Parkkari J, Sievänen H, Heinonen A, Vuori I, Järvinen M (1996). Epidemiology of hip fractures. Bone.

[5] Court-Brown C, Heckman JD, McKee M, McQueen MM, Ricci W, Tornetta P (2015). Rockwood and Green's Fracture in Adults.

[6] van Embden D, Rhemrev SJ, Meylaerts SA, Roukema GR (2010). The comparison of two classifications for trochanteric femur fractures: The AO/ASIF classification and the Jensen classification. Injury.

[7] Ravinath TM, Jeyaraman M, Chaudhari K, Ravi Weera AV, Sabarish K, Selvaraj P (2019). Surgical Management of Proximal Femoral Fractures by Proximal Femoral Nailing-An Institutional Experience. Ortho Res Online J.

[8] Kim JW, Kim TY, Ha YC, Lee YK, Ko KH (2015). Outcome of intertrochanteric fractures treated by intramedullary nail with two integrated lag screws: A study in Asian population. Indian J Orthop.

[9] Laohapoonrungsee A, Arpornchayanon O, Phornputkul C (2005). Two-hole side-plate DHS in the treatment of intertrochanteric fracture: Results and complications. Injury.

[10] Haidukewych GJ (2009). Intertrochanteric Fractures: Ten Tips to Improve Results. J Bone Joint Surg Am.

[11] Audigé L, Bhandari M, Kellam J (2004). How reliable are reliability studies of fracture classifications? A systematic review of their methodologies. Acta OrthopaedicaScandinavica.

[12] Landis JR, Koch GG (1977). The Measurement of Observer Agreement for Categorical Data. Biometrics.

[13] Pervez H1, Parker MJ, Pryor GA, Lutchman L, Chirodian N (2002). Classification of trochanteric fracture of the proximal femur: a study of the reliability of current systems. Injury.

[14] Shen J, Hu FK, Zhang LH, Tang PF, Bi ZG (2013). Preoperative classification assessment reliability and influence on the length of intertrochanteric fracture operations. International Orthopaedics.

[15] Dai LY, Jin WJ (2005). Interobserver and intraobserver reliability in the load sharing classification of the assessment of thoracolumbar burst fractures. Spine.

[16] Schipper IB, Steyerberg EW, Castelein RM, van Vugt AB (2001). Reliability of the AO/ASIF classification for pertrochanteric femoral fractures. Acta OrthopaedicaScandinavica.

[17] De Boeck H (1994). Classification of hip fractures. Acta orthopaedicaBelgica.

[18] Newey ML, Ricketts D, Roberts L (1993). The AO classification of long bone fractures: an early study of its use in clinical practice. Injury.

